# MyD88 is crucial for the development of a protective CNS immune response to *Toxoplasma gondii* infection

**DOI:** 10.1186/1742-2094-10-19

**Published:** 2013-02-01

**Authors:** Marbel Torres, Rachel Guiton, Sonia Lacroix-Lamandé, Bernhard Ryffel, Samuel Leman, Isabelle Dimier-Poisson

**Affiliations:** 1 Université de Tours, UMR1282 Infectiologie et Santé Publique, UFR Pharmacie, Tours, F-37000, France; 2 Institut National de la Recherche Agronomique (INRA), UMR1282 Infectiologie et Santé Publique, Immunologie Parasitaire, Vaccinologie et Biothérapies Anti-Infectieuses, Nouzilly, F-37380, France; 3 INRA, UMR1282 Infectiologie et Santé Publique, Nouzilly, F-37380, France; 4 Université de Tours, UMR1282 Infectiologie et Santé Publique, Contrôle et Immunologie des Maladies Entériques du Nouveau-né, Tours, F-37000, France; 5 INEM-UMR7355, 6218 Université-Centre National de la Recherche Scientifique (CNRS), Immunologie et Embryologie Moléculaire, Institut de Transgénose, 3B rue de la Férollerie, Orléans, 45071, France; 6 UMR INSERM U930, Université François Rabelais, Imagerie et Cerveau, Equipe 4: Troubles Affectifs, Unité de Formation et de Recherche (UFR) Sciences et Techniques, Parc Grandmont, Tours, 37200, France

**Keywords:** MyD88, innate immunity, *Toxoplasma gondii*, BALB/c mice, encephalitis

## Abstract

**Background:**

Toxoplasmosis is one of the most common parasitic infections in humans. It can establish chronic infection and is characterized by the formation of tissue cysts in the brain. The cysts remain largely quiescent for the life of the host, but can reactivate and cause life-threatening toxoplasmic encephalitis in immunocompromised patients, such as those with AIDS, neoplastic diseases and organ transplants. Toll-like receptor (TLR) adaptor MyD88 activation is required for the innate sensing of *Toxoplasma gondii*. Mice deficient in MyD88 have defective IL-12 and Th1 effector responses, and are highly susceptible to the acute phase of *T. gondii* infection. However, the role of this signaling pathway during cerebral infection is poorly understood and requires examination.

**Method:**

MyD88-deficient mice and control mice were orally infected with *T. gondii* cysts*.* Cellular and parasite infiltration in the peripheral organs and in the brain were determined by histology and immunohistochemistry. Cytokine levels were determined by ELISA and chemokine mRNA levels were quantified by real-time PCR (qPCR).

**Results:**

Thirteen days after infection, a higher parasite burden was observed but there was no histological change in the liver, heart, lungs and small intestine of MyD88^−/−^ and MyD88^+/+^ mice. However, MyD88^−/−^ mice compared to MyD88^+/+^ mice were highly susceptible to cerebral infection, displayed high parasite migration to the brain, severe neuropathological signs of encephalitis and succumbed within 2 weeks of oral infection. Susceptibility was primarily associated with lower expression of Th1 cytokines, especially IL-12, IFN-γ and TNF-α, significant decrease in the expression of CCL3, CCL5, CCL7 and CCL19 chemokines, marked defect of CD8^+^ T cells, and infiltration of CD11b^+^ and F4/80^+^ cells in the brain.

**Conclusion:**

MyD88 is essential for the protection of mice during the cerebral installation of *T. gondii* infection. These results establish a role for MyD88 in T cell-mediated control of *T. gondii* in the central nervous system (CNS).

## Background

*Toxoplasma gondii* is a protozoan parasite responsible for toxoplasmosis, a worldwide disease that infects approximately one-third of the world’s population. Despite the development of protective immunity, parasites persist in latent cyst form in multiple tissues, most prominently in the brain. In immunocompetent individuals, the chronic phase is asymptomatic, but leads to toxoplasmic encephalitis in immunocompromised individuals [1]. During primary acute infection, *T. gondii* induce a protective immunity, which is thought to mainly involve a systemic Th1 cellular immune response, which is focused on IL-12 secreted by dendritic cells (DCs) and IFN-gamma production by CD4^+^ and CD8^+^ T lymphocytes [2]. Recent research on the molecular signaling pathways triggered during the infection of *T. gondii* have demonstrated that the adaptor molecule MyD88, recruited after Toll-like receptor (TLR) activation by parasite molecules, is required for the establishment of this protective immune response.

Studies carried out in MyD88^−/−^ mice unambiguously demonstrate that this adaptor molecule is required to induce a protective immune response against *T. gondii* infection. The implication of MyD88 during the immune response against *T. gondii* was first shown by Scanga *et al.*[3]. Using MyD88-deficient C57BL/6 mice infected intraperitoneally with an avirulent strain of *T. gondii*, they demonstrated that MyD88 was essential for the survival to the acute phase of infection and for early parasite replication control. Moreover, such mice are unable to produce IL-12, a major cytokine in the setting of an efficient Th1 response [3]. More recent work confirmed these results following infection of the same type of mice via the oral route. MyD88^−/−^ mice displayed early death associated with uncontrolled parasite replication. IL-12 response was severely curtailed during oral infection [4]. Taken together, these data suggest that optimal resistance to *T. gondii* involves responses to MyD88. There is also evidence that TLR signaling is strongly implicated, since tachyzoite profilin and glycosylphosphatidylinositol (GPI) have recently been identified as parasite ligands of TLR11 and TLR2/4 respectively [5-7].

Once infection progresses to the chronic phase, it is crucial to determine the implication of MyD88 in the process to limit parasite replication in the brain. For the first time, we used MyD88-deficient mice in resistant BALB/c background in an experimental *T. gondii* infection, to assess the role of the TLR adaptor protein MyD88 for host resistance to *T. gondii* cerebral infection. The studies presented here demonstrate that while MyD88-deficient mice are able to control the early phase of infection, they were highly susceptible to cerebral infection. This susceptibility is correlated to the brain of MyD88 KO animals with a significant reduction in the expression of several inflammatory mediators and in CD8^+^ T cells, a significant infiltration of CD11b^+^ and F4/80^+^ cells associated with an increased parasite migration to and replication in the brain. MyD88 plays an essential role in establishing the protective central nervous system (CNS) host response during the installation of *T. gondii* infection in the brain.

## Materials and methods

### Mice

MyD88-deficient mice (MyD88^−/−^), obtained from Dr S Akira (Research Institute for Microbial Disease, Osaka University, Japan) were backcrossed 10 times on the wild-type BALB/c background and bred at the Institute de Transgénose (CNRS, Orleans, France) under specific pathogen free (SPR) conditions. Age-matched BALB/c mice (MyD88^+/+^) were purchased from Janvier (Saint Berthevin, France) and used as controls in all experiments. Adult females weighing 20 to 25 g and aged 10 to 12 weeks were orally infected with 80 cysts of the 76 K strain, obtained as described below. In some experiments, mice were treated each day with sulfadiazine in their drinking water (400 mg/l), beginning 4 days after infection.

The animal experiments complied with the French Government’s ethical and animal experiment regulations.

### Parasites and *T. gondii* extract

Tachyzoites of the RH strain of *T. gondii* were harvested from infected monolayers of human foreskin fibroblast (HFF) Hs27 (ATCC CRL-1634) and were used as a source of *T. gondii* extract [8].

Cysts of the 76 K strain of *T. gondii* were obtained from the brains of CBA/J mice that had been orally infected with 50 cysts 1 month earlier.

### Antigen-induced cytokine production in culture

Spleens and mesenteric lymph nodes (MLNs) were harvested 13 days after the infection and pressed through a stainless steel mesh. Single cell suspensions were obtained by filtration through a nylon mesh to remove tissue debris. Spleen erythrocytes were lysed by hypotonic shock, single cells of spleen, and MLNs were resuspended in RPMI 1640 supplemented with 5% FCS, HEPES (25 mM), L-glutamine (2 mM), sodium pyruvate (1 mM), β-mercaptoethanol (5 × 10^-5^ M) and penicillin (50 U/ml)/streptomycin (50 μg/ml).

Spleen and MLN cells were cultured in 24-well plates at 10^6^ cells per well, in 1 ml of culture medium, alone or containing *Toxoplasma* extract (10 μg/ml) [8]. The plates were incubated for 4 days in 5% CO_2_ at 37°C. Cell culture supernatants were harvested between 24 hours and 4 days, and kept at −20°C until cytokine ELISA.

Spleen and MLN cell supernatants were tested for IL-2, IL-4, IL-10, IL-5, IL-13, IFN-γ, IL-12 and nitric oxide (NO) activities. Cytokine productions were evaluated using commercial ELISA kits according to the manufacturer’s instructions (e-Bioscience, San Diego, CA, USA), except for IL-12 p40 (DuoSet, R&D Systems, Minneapolis, MN, USA). Concentrations were determined by reference to standard curves constructed with known amounts of mouse recombinant IL-2, IL-4, IL-10, IL-5, IL-13 and IFN-γ. The sensitivity limits for the assays were 3.1 pg/ml for IL-2, 7.8 pg/ml for IL-4, 15.6 pg/ml for IL-5, and 31.3 pg/ml for IL-10, IL-13, IL-12p 40 and IFN-γ.

To test the presence of pro- and anti-inflammatory cytokines in the brains of infected animals, brains were harvested 13 days after infection and homogenized in 5 ml of RPMI 1640 with a pestle and mortar. Organs were then centrifuged for 5 minutes at 10,000 × g and the supernatants were tested for the presence of IFN-γ, IL-12p 40, IL-10, IL-2 and IL-13 by ELISA, as described above.

The NO production was evaluated using the Griess method. Briefly, Griess reagents (sulfanimide 1% and naphtylethylenediamine 0.1%, ratio 1:1; Sigma-Aldrich, St Louis, MO, USA) were added to the culture supernatants. The absorbance was measured at 540 nm. The NO concentration was determined with a standard curve established with a 20 nM sodium nitrite solution. The sensitivity limit was 2 μM.

### RNA extraction

MLNs, spleen, liver, heart, lungs, small intestines and brains were removed from infected and uninfected mice 13 days postinfection. The organs were placed in TRIzol (Gibco-BRL, Life Technologies, Grand Island, NY, USA) and were crushed with an ULTRA-TURRAX (IKA, Staufen, Germany). The samples were centrifuged at 8,000 x g to eliminate debris and then the supernatants were stored at −80°C until further processing. RNA was extracted according to the manufacturer’s instructions until the isopropanol addition step. The mixes were then added on Qiagen mini columns (Valencia, CA, USA) and spun for 15 seconds at 8,000 x g. The flow-through was discarded and the columns were washed. The following steps were carried out according to the manufacturer’s instructions. RNA quality was estimated by agarose gel electrophoresis using ethidium bromide for staining and RNA quantification was performed by Nanodrop (Thermo Fisher Scientific, Courtaboeuf, France) measuring absorbance at 260 nm.

### Analyses of inflammatory chemokines and cytokines by real-time reverse-transcription PCR (qRT-PCR)

Total RNA was reverse transcribed using oligo(dT) primers and M-MLV reverse transcriptase (Promega, Madison, WI, USA) according to the manufacturer’s instructions. Synthesized cDNA was then amplified by PCR using Chromo4 apparatus (Bio-Rad, Hercules, CA, USA). Hypoxanthine phosphoribosyltransferase mRNA levels were used to normalize RNA quantification. We used various primers for chemokine (CCL3, CCL5, CCL7, CCL19, CCL20 and CCL21) [9], cytokine (IL-17 and IL23) [10] and stage-specific *Toxoplasma* antigen (tachyzoite (SAG1) and bradyzoite (BAG1)) [11] mRNA quantification. All real-time PCR (qPCR) displayed an efficiency of between 90% and 110%. Diluted cDNA was combined with primers and iQ SYBR Green Supermix (Bio-Rad) according to the manufacturer’s recommendations, and real-time assays were run on a Chromo4 (Bio-Rad). The specificity of the qPCR reactions was assessed by analyzing the melting curves of the products and size verification of the amplicons. The information was processed to calculate the relative gene expression values determined according to a standard curve.

### Histology and immunohistochemistry

For CD8α, CD3 and F4/80 fluorescence staining, at day 13 postinfection, brains were removed and embedded in optimal cutting temperature (OCT) Tissue Tek compound (Miles Scientific, Naperville, IL, USA) before storage at −80°C until use. Frozen sections of the brains (8 μm thick) were performed with a cryostat (Leica CM3050S, Leica Microsystems, Buffalo Grove, IL, USA), fixed in acetone gradient and incubated with primary antibodies (rat monoclonal antibodies anti-CD8α, anti-CD3 and F4/80; AbD Serotec, Kidlington, UK) or a rabbit serum of *Toxoplasma* infection for 2 hours at room temperature. Sections were washed several times in PBS and were then incubated with a donkey anti-rat IgG Alexa Fluor 594 (Life Technologies) for 1 hour at 37°C or a donkey anti-rabbit FITC (DAKO, Glostrup, Denmark). Sections were counterstained with Hoechst nuclear dye, mounted in Vectashield (H1000, Vector Laboratories, Burlingame, CA, USA) and observed on a Zeiss fluorescence microscope with Axiovision software (Zeiss, Göttingen, Germany).

For CD11b fluorescence staining, adjacent coronal sections of the brains from infected MyD88^−/−^ and MyD88^+/+^ mice were stained. Briefly, after deep anesthesia (sodium pentobarbital, 40 mg/kg, intraperitoneal (i.p.)), mice were perfused through the heart with 80 ml of saline followed by 200 ml of 4% paraformaldehyde in 0.1 M PBS (pH 7.4). Brains were removed, postfixed for 2 hours in the same fixative and cryoprotected in a 20% sucrose solution overnight at 4°C. Coronal sections (45 μm thick) were cut with a cryostat (Leica CM3050S) and collected every four sections. After a series of washes in 50% ethanol and 3% H_2_O_2_, free floating sections were incubated at room temperature in primary rat anti-mouse CD11b antibody (clone 5C6, 1:500; AbD Serotec), in 5% normal horse serum, 0.2% Triton X-100 (Sigma-Aldrich) in PBS. Thirty-six hours later, sections were washed in 0.1 M PBS, incubated for 2 hours in a biotinylated anti-rat IgG (1:500; Jackson ImmunoResearch, West Grove, PA, USA) followed by ABC Kit (1:100, 1 hour; Vector Laboratories) and reacted with 3,3’-diaminobenzidine (DAB; Sigma-Aldrich) in the presence of H_2_O_2_. Sections were rinsed, mounted on gelatinized glass slides, dehydrated, cleared in Claral and coverslipped with Eukitt (Sigma-Aldrich).

### Cyst counts in the brain

Thirteen days after infection, brains were harvested from surviving mice and homogenized in 5 ml of RPMI 1640 with a pestle and mortar. The cysts in each brain homogenate were counted under a microscope (10 counts, each on 10 μl). The results are expressed as means ± standard error of the mean (SEM) for each group.

### Real-time PCR (qPCR) in the brain to assess parasite load

A total of 1 μg genomic DNA was prepared from the brain using a DNeasy tissue kit (Qiagen) according to the manufacturer’s instructions. Parasite burden was measured by amplifying the *T. gondii* B1 gene using the 2 × SYBR Green PCR Master Mix (Life Technologies) on an iCycler (Bio-Rad).

The primers used for QB1 were: forward, 5^′^-GGAACTGCATCCGTTCATGAG-3^′^; reverse, 5^′^-TCTTTAAAGCGTTCGTGGTC-3^′^. A standard curve for parasite equivalents was generated using a plasmid, as described previously [12].

### Statistical analysis

Statistical analysis was performed using the Mann–Whitney U test (GraphPad Prism software, La Jolla, CA, USA) to analyze the observed differences in cytokines, chemokines and cyst counts. A *P* value <0.05 was considered significant.

## Results

### MyD88^−/−^ BALB/c mice are highly susceptible to cerebral *T. gondii* infection

**Figure 1:**
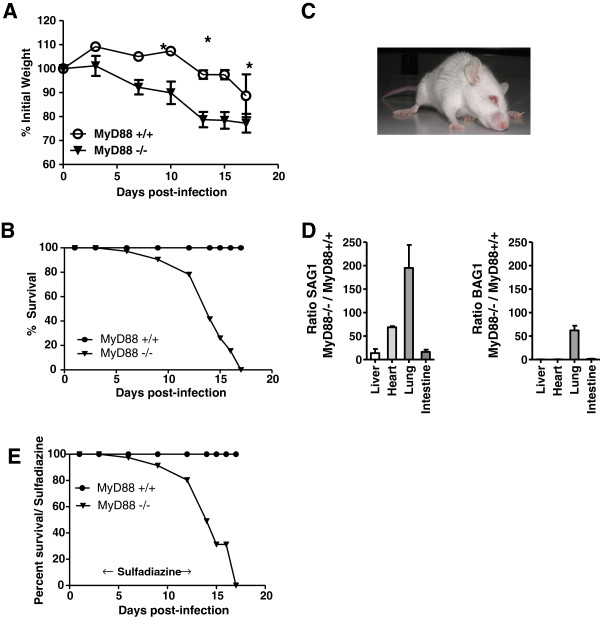
**MyD88^−/−^ BALB/c mice are highly susceptible to oral infection with *T. gondii.*** (**A**) Loss of weight after oral infection with 80 cysts of the 76 K *T. gondii* strain. The total number of tested animals in each group is n = 7. This result is representative of three independent experiments. (**B**) Survival after oral infection with 80 cysts of the 76 K *T. gondii* strain. Data are expressed as percentage of cumulative survival during the experiment. The total number of tested animals in each group is n = 7. This result is representative of three independent experiments. (**C**) Picture of a MyD88^−/−^ BALB/c mouse with neurotoxoplasmosis symptoms at 13 days postinfection displaying a tilted position. (**D**) Expression of bradyzoite (BAG1) and tachyzoite (SAG1) mRNA in the lungs, heart, liver and small intestine of MyD88^−/−^ and MyD88^+/+^ BALB/c mice 13 days postinfection. The results are presented as a ratio of the expression level in MyD88^−/−^ mice on the expression in MyD88^+/+^ mice. Results are representative of two different experiments. The total number of tested animals in each group are n = 7. (**E**) Survival after oral infection with 80 cysts of the 76 K *T. gondii* strain of sulfadiazine-treated mice. Mice were treated with sulfadiazine beginning 4 days after infection. The total number of tested animals in each group is n = 6. This result is representative of two independent experiments.

To evaluate the contribution of MyD88 signaling in the induction of protective immune response against *T. gondii* on genetically resistant BALB/c background, MyD88^+/+^ and MyD88^−/−^ BALB/c mice were orally infected with 80 cysts of the 76 K strain of *T. gondii*. Their survival was monitored over 30 days and they were weighed on a daily basis over the experimental time course as an indicator of morbidity. From day 9 to 17, infected MyD88^−/−^ mice showed greater weight loss than MyD88^+/+^ mice (Figure 1A), and displayed other severe symptoms including persistent hunched posture, unresponsiveness to provocation and lack of interaction with littermates. A decrease in autonomic nervous system function of infected MyD88^−/−^ mice, measured by increased urination and defecation, was also statistically significant when compared to infected MyD88^+/+^ mice (data not shown). Within 17 days of infection, while MyD88^+/+^ mice survive, all MyD88^−/−^ mice succumb (Figure 1B), presenting signs of neurotoxoplasmosis characterized by a tilted head to one side and circling in the cage (Figure 1C), whereas MyD88^+/+^ mice did not show any clinical symptoms.

Taken together, these results indicated that the susceptibility of MyD88^−/−^ BALB/c mice was due to an increased infectivity in the brain. To confirm this hypothesis, and since mortality during acute *T. gondii* infection can result from either uncontrolled inflammation [13] or increased parasite replication [14,15], we assessed parasite burden in different organs (small intestine, liver, heart, lungs and brain), and analyzed immune response in the spleen, MLNs and brain.

### Mesenteric lymph nodes (MLNs) from MyD88^−/−^ mice show impaired cellular response

To determine why MyD88^−/−^ mice exhibited a profound deficiency in their ability to control *Toxoplasma* brain infection, mucosal immune responses were analyzed in MLNs.

**Figure 2:**
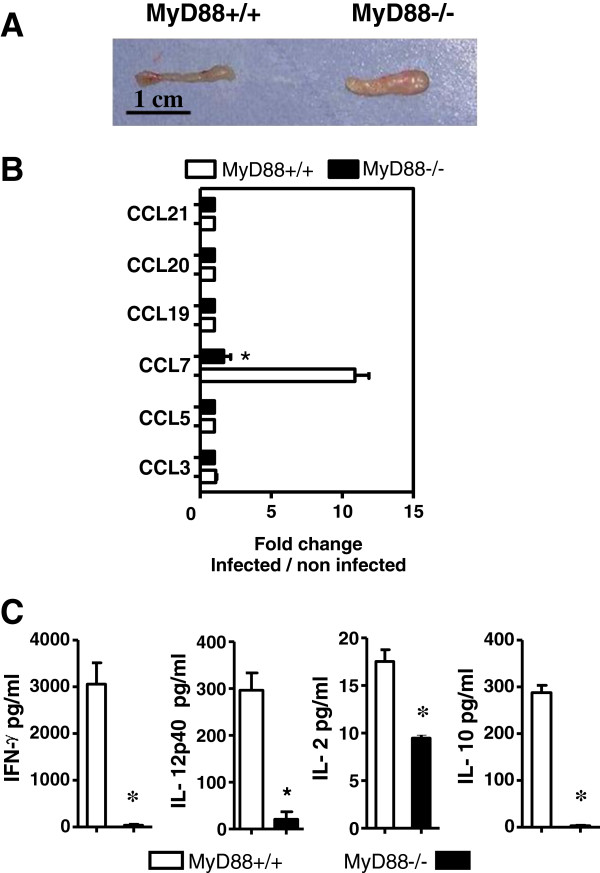
**Impaired mucosal immune response in MyD88^−/−^mice infected with *T. gondii.*** (**A**) Macroscopic observation of MyD88^+/+^ and MyD88^−/−^ MLNs of mice infected with 80 cysts of the 76 K *T. gondii* strain 13 days postinfection. (**B**) qRT-PCR evaluation of the expression of chemokine mRNA in MLNs of MyD88^+/+^ (open bars) and MyD88^−/−^ (solid bars) BALB/c mice 13 days postinfection. Results are presented as the fold increase expression of mRNA in infected mice versus expression of mRNA in non-infected mice. The results are representative of two experiments with 7 mice per group. (**C**) cytokine levels in supernatants of MLN cells restimulated by *Toxoplasma* extract from MyD88^+/+^ and MyD88^−/−^ BALB/c mice 13 days postinfection. Supernatants were collected between 48 and 96 hours to determine cytokines levels (IL-10 at 72 h, IL-2 at 48 h, IFN-γ at 96 h and IL-12 p40 at 48 h) in MyD88^+/+^ (open bars) and MyD88^−/−^ (solid bars) mice. These results are representative of two independent experiments with 7 mice per group. Data analyzed using a Mann–Whitney test * (*P* <0.05). MLN, mesenteric lymph node; qRT-PCR, real-time reverse-transcription polymerase chain reaction.

Thirteen days postinfection, MLNs of the sacrificed mice were removed for cellular analyses. MLNs from MyD88^+/+^ mice appeared macroscopically to have a classical size and morphology, whereas MLNs from the MyD88^−/−^ mice were enlarged with a granular surface (Figure 2A). However, no difference in cell numbers of MLNs was observed between the MyD88^+/+^ and MyD88^−/−^ mice (data not shown). Since secretion of chemokines is responsible for the inflammatory cell trafficking toward the sites of infection and the recruitment of immune cells, such as macrophages, dendritic cells and neutrophils, we measured mRNA levels of various chemokines such as CCL3, CCL5, CCL7, CCL19, CCL20, CCL21, by real-time reverse-transcription PCR (qRT-PCR) analysis. Except for CCL7, there was no upregulation of mRNA expression of chemokines after infection in both MyD88^−/−^ and MyD88^+/+^ mice (Figure 2B).

MyD88 signaling has been reported to be required for the production of certain cytokines by innate immune cells such as DCs, macrophages and neutrophils. Therefore, we then stimulated the MLN cells with *T. gondii* protein extract, and evaluated the cytokine (IFN-γ, IL-12, IL-2, IL-4, IL-5, IL13 and IL-10) and NO production in the culture supernatants. The IFN-γ, IL-12, IL-2 and IL-10 secretions were dramatically diminished in MyD88^−/−^ mice compared to the MyD88^+/+^ animals: 37 and 3059 pg/ml of IFN-γ respectively (*P* <0.05), 20 and 296 pg/ml of IL-12 (*P* <0.05), 9 and 17 pg/ml of IL-2 (*P* <0.05), and 3 and 287 pg/ml of IL-10 (*P* <0.05) (Figure 2C). NO levels were not significantly different between MyD88^−/−^ and MyD88^+/+^ mice (0.8 and 0.4 μM respectively). The presence of IL-4, IL-5 and IL13 could not be detected in the supernatants (data not shown).

In the intestinal compartment, MLNs of MyD88^−/−^ mice showed an absence of efficient cytokine response against *T. gondii* 13 days postinfection.

### Spleens from MyD88^−/−^ mice show impaired cytokine response

**Figure 3:**
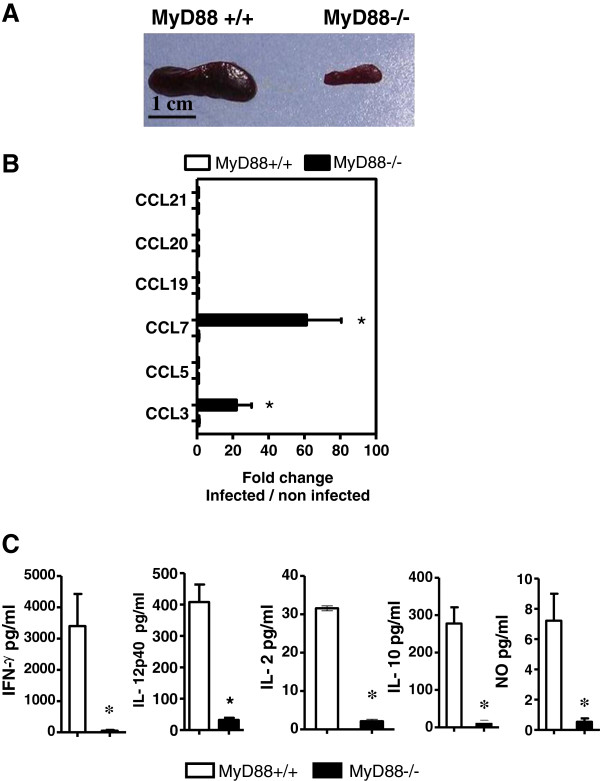
**Impaired splenic immune response in MyD88^−/−^mice infected with *T. gondii.*** (**A**) Splenomegaly in MyD88^−/−^ but not in MyD88^+/+^ mice, infected with 80 cysts of the 76 K *T. gondii* strain 13 days postinfection. (**B**) Expression of chemokine mRNA in the spleens of MyD88^+/+^ (open bars) and MyD88^−/−^ (solid bars) BALB/c mice 13 days postinfection, evaluated by qRT-PCR. Results are presented as the fold increase expression of mRNA in infected mice versus expression of mRNA in non-infected mice. The results are representative of two independent experiments with 7 mice per group. (**C**) cytokine and NO production by *Toxoplasma* extract stimulated spleen cells from MyD88^−/−^ and MyD88^+/+^ BALB/c mice 13 days postinfection. Supernatants were collected between 24 and 96 hours to determine cytokines levels (IL-10 at 72 hours, IL-2 at 48 hours, IFN-γ at 96 hours, IL-12 p40 at 48 hours and NO at 24 hours) in MyD88^+/+^ (open bars) and MyD88^−/−^ (solid bars) mice. These results are representative of two independent experiments. The total number of tested animals in each group is n = 7. Data analyzed using a Mann–Whitney test * (*P* <0.05). qRT-PCR, real-time reverse-transcription polymerase chain reaction.

Thirteen days after infection, MyD88^−/−^ and MyD88^+/+^ mice were sacrificed, and their spleens were removed for cellular analysis. Macroscopically, spleens from MyD88^−/−^ mice failed to display the splenomegaly observed in infected MyD88^+/+^ controls (Figure 3A), suggesting an impaired immune response in those mice. To characterize the immune response in the spleens of *T. gondii*-infected BALB/c MyD88^+/+^ and MyD88^−/−^ mice, mRNA levels of chemokines were measured by qRT-PCR analysis. Thirteen days after infection, mRNA expression of CCL3 and CCL7 in the spleens of MyD88^−/−^ was increased by approximately 21 and 61 fold respectively over non-infected MyD88^−/−^. These over-expressions were significantly higher than those observed in MyD88^+/+^ mice (Figure 3B). Conversely, there was no significant increase in mRNA expression of CCL5, CCL19, CCL20 and CCL21 chemokines after infection in both MyD88^+/+^ and MyD88^−/−^ mice (Figure 3B). As in MLN cells, cytokine production and NO synthesis were measured in the supernatant of *T. gondii*-extract-stimulated-splenocytes. Cytokines such as IL-17 and IL-23 were analyzed by qRT-PCR. No significant difference was observed between the two genotypes (data not shown). The production of IFN-γ, IL-12, IL-2, IL-10 and NO was dramatically decreased in MyD88^−/−^ mice compared to their wild-type counterparts: 54 and 3404 pg/ml of IFN-γ respectively (*P* <0.05), 32 and 408 pg/ml of IL-12 (*P* <0.05), 1 and 31 pg/ml of IL-2 (*P* <0.05), 11 and 278 pg/ml of IL-10 (*P* <0.05), and 0.5 and 7 pg/ml of NO (*P* <0.05) (Figure 3C). We could not find any significant level of IL-4, IL-5 and IL-13 in the supernatants of the cultured spleen cells from either MyD88^+/+^ or MyD88^−/−^ mice. These results suggest a dramatic reduction of innate and adaptive immune responses in the spleens of mice deficient in MyD88 adaptor molecule.

### Higher parasite burden but no histological change in peripheral organs of MyD88^−/−^ mice compared to MyD88^+/+^ mice

Using qPCR, the mRNA levels of BAG1 and SAG1 were measured to analyze the replication of tachyzoites and bradyzoites in the liver, lungs, heart and small intestine. We observed a moderate increase in the expression of SAG1 in the liver and the small intestine (approximately 13 and 16 fold respectively), but an important increase in the heart and lungs of MyD88^−/−^ mice infected with *T. gondii* compared to MyD88+/+ mice (68 and 195 fold respectively) (Figure 1D). In addition, bradyzoites were not detectable in peripheral organs of MyD88^+/+^ and MyD88^−/−^ mice, except in the lungs of MyD88^−/−^ mice (Figure 1D).

After histopathological analysis of these different organs, we observed that no groups exhibited any pathology in the liver and small intestine. Although there were no significant differences between MyD88^+/+^ or MyD88^−/−^ mice, the two groups exhibited limited indicators of pathology with a mild inflammation of the heart and lungs (data not shown). Moreover, MyD88^−/−^ mice organs did not have significantly greater extracellular parasite burden than MyD88^+/+^ mice organs. In addition, there were no significant differences in *T. gondii*-specific IgG1 and IgG2a antibody levels or serum levels of IL-4, IL-12 or IFN-γ between MyD88^+/+^ or MyD88^−/−^ mice (data not shown).

In order to determine if the mortality observed in infected MyD88^−/−^ mice after the second week of infection was associated with toxoplasmic myocarditis, pneumonia and/or encephalitis, a sulfadiazine treatment was given to these animals and to MyD88^+/+^ mice every day beginning 4 days after infection. Surprisingly, MyD88^−/−^ mice were unable to establish a chronic infection and all succumbed during the sulfadiazine treatment from day 13 to day 17 (Figure 1E). Once more, MyD88^−/−^ mice revealed symptoms of an infection at the cerebral level with piloelection, huddling, lost of mobility, a tilted head to one side and circling in the cage, indicating a persistence of tachyzoite proliferation in the brain. In contrast, control-infected mice did not develop illness and all survived until the end of the observation period. In MyD88^−/−^ mice, active infection with tachyzoites appears to continue in the brain during the entire period of sulfadiazin treatment.

These results suggest that the brains of MyD88^−/−^ mice were highly parasitized by tachyzoite forms of *T. gondii*.

### MyD88^−/−^ mice show increased brain pathology during infection with *T. gondii*

**Figure 4:**
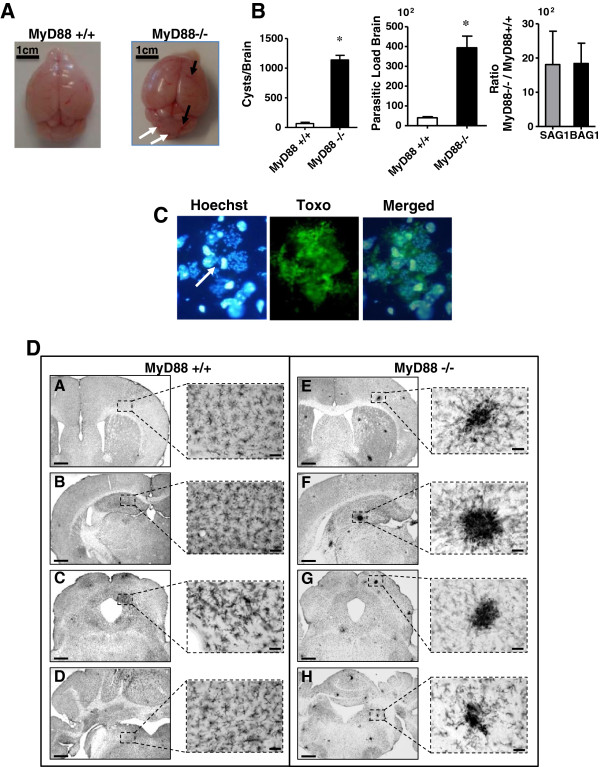
**The particular case of the brain. **(**A**) Macroscopic observation of the brains of MyD88^−/−^ and MyD88^+/+^ mice infected with 80 cysts of the 76 K *T. gondii* strain 13 days postinfection. Brains from MyD88^−/−^ mice revealed distinct hemorrhagic (black arrows) and necrotic areas (white arrows), differing substantially from MyD88^+/+^ controls. (**B**) Thirteen days postinfection, mice from both genotypes were sacrificed and their brains were removed to analyze parasite loads after brain homogenization by microscopic counting (left graph), by qPCR (middle graph), and by qRT-PCR to analyze expression of bradyzoite (BAG1) and tachyzoite (SAG1) mRNA (right graph). The results are presented as the ratio of the expression level in MyD88^−/−^ mice to the expression in MyD88^+/+^ mice. The data were statistically analyzed using the Mann–Whitney U test (GraphPad Prism software), * (*P* <0.05). The results are representative of four independent experiments with n = 7 mice. (**C**) Immunofluorescence staining of *Toxoplasma gondii* tachyzoites in the brain of MyD88^−/−^ mice. Frozen brain sections were stained with a polyclonal rabbit anti-*Toxoplasma*, followed by a FITC-conjugated donkey anti-rabbit serum and counterstained with Hoechst to show nuclei. The arrow shows DNA parasite (x 100). (**D**) Microglia was stained using a CD11b antibody. (**A**) to (**D**): microphotographs of the wild-type MyD88^+/+^ only showing ramified microglia and very few clustered ameboid microglia; (**E**) to (**H**): microphotographs of MyD88^−/−^ brain sections showing numerous clustered ameboid and ramified microglia. (**A**) and (**E**): striatum and cortex areas; (**B**) and (**F**): hippocampal area; (**C**) and (**G**): periaqueductal gray and midbrain areas; (**D**) and (H): brainstem and cerebellum. The staining was repeated in two other mice in each group with similar staining. Bar = 500 μm left column, bar = 50 μm right column. FITC, fluorescein isothiocyanate; qPCR, real-time polymerase chain reaction; qRT-PCR, real-time reverse-transcription polymerase chain reaction.

Macroscopic observation of brains from MyD88^−/−^ mice revealed distinct hemorrhagic and necrotic areas, differing substantially from MyD88^+/+^ controls (Figure 4A).

MyD88^−/−^ mice had significantly more cysts than MyD88^+/+^ mice (1137 versus 65 cysts per brain respectively, *P* <0.05) (Figure 4B, left graph). Thirteen days after infection, quantification of parasite DNA in the brain revealed an increased parasite burden by sevenfold (40,000 versus 5,700 parasitic burden) (Figure 4B, middle graph). Moreover, using qPCR the mRNA levels of BAG1 and SAG1 were measured and analyzed to distinguish the replication of tachyzoites and bradyzoites (Figure 4B, right graph). We observed a strong increase of SAG1 and BAG1 expression in the brains of MyD88^−/−^ mice infected with *T. gondii* compared to MyD88^+/+^ mice (about 1,800 fold for SAG1 and BAG1). These results suggest that brains of MyD88^−/−^ mice were highly parasitized by tachyzoite and bradyzoite forms of *T. gondii*.

*Toxoplasma* immunolabeling indicated that the most prominent pathology was evident in the brain, with the presence of a high number of extracellular parasites 13 days postinfection of MyD88^−/−^ mice (Figure 4C).

Using immunohistochemical and histopathological methods on sections of the brain, we examined the inflammatory cell responses in the brains of MyD88^−/−^ and MyD88^+/+^ mice, by immunostaining for CD11b, F4/80, CD8α and CD3. In infected MyD88^−/−^ mice, CD11b labeling showed widespread macrophage/microglial activation observed all over the brain from the olfactory bulbs to the brainstem levels. In particular, the immunolabeling showed numerous clusters of intensely stained ameboid microglia, which are illustrated in Figure 4D.

**Figure 5:**
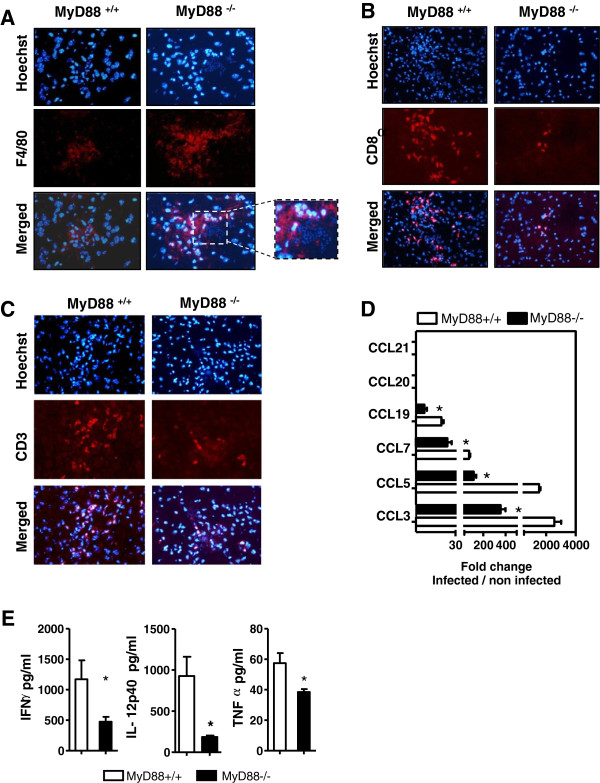
**Enhanced neurotoxoplasmosis.** Immunofluorescence staining of (**A**) F4/80, (**B**) CD8α and (**C**) CD3^+^ cells in the brains of MyD88^−/−^ and MyD88^+/+^ mice. Frozen brain sections were stained with rat anti-F4/80, rat anti-CD8 and rat anti-CD8, followed by an Alexa 594-conjugated secondary antibody against rat IgG. Sections were counterstained with Hoechst (x 100). (**D**) Expression of chemokine mRNA in the brains of MyD88^+/+^ (open bars) and MyD88^−/−^ (solid bars) BALB/c mice 13 days postinfection evaluated by qRT-PCR. Results are presented as the fold increase expression of mRNA in infected mice versus expression of mRNA in non-infected mice. The results are representative of two different experiments. The total number of tested animals in each group is n = 7. (**E**) Determination of cytokine levels in supernatants of brain homogenates from MyD88^+/+^ and MyD88^−/−^ BALB/c mice 13 days postinfection. Supernatants from MyD88^+/+^ (open bars) and MyD88^−/−^ (solid bars) were collected to determine cytokine levels. Results are representative of two experiments and were analyzed using a Mann–Whitney test. * (*P* <0.05). qRT-PCR, real-time reverse-transcription polymerase chain reaction.

In contrast, ramified microglia are evenly distributed throughout the brain of infected MyD88^+/+^ mice with only very few clustered microglia. No clusters were observed in non infected MyD88^+/+^ mice (data not shown). Immunofluorescence staining with F4/80 gave similar results as CD11b immunolabeling. In addition, parasites could be observed on the brain section with Hoechst stain within F4/80^+^ cells (Figure 5A).

We observed an increase of CD8α (Figure 5B) and CD3^+^ cells (Figure 5C) in MyD88^+/+^ mice compared to MyD88^−/−^ mice, suggesting an infiltrate of T lymphocytes in the brains of infected MyD88^+/+^ mice.

It has been shown that chemokines are important in controlling *T. gondii* infection and ensuing encephalitis, in part due to their ability to recruit immune effector cells to sites of infection. After infection, mRNA transcript levels of CCL3, CCL5, CCL7 and CCL19 were significantly and consistently increased in the brains of MyD88^+/+^ mice compared to the brains of MyD88^−/−^ mice (about 2,500 fold for CCL3; 1,500 fold for CCL5; 70 fold for CCL7; and 20 fold for CCL19, compared to non-infected) (Figure 5D). The expression of those transcripts was much lower in MyD88^−/−^ mice. Taken together, these results showed that the chemokine mRNA responses in the brains of infected mice were significantly different between MyD88^−/−^ and MyD88^+/+^ mice. In contrast, transcript levels of CCL20, CCL21 were detected at similar levels in infected MyD88^+/+^ and MyD88^−/−^ mice. There is no increase of mRNA expression for IL-17 and IL-23 in the brains of infected MyD88^−/−^ and MyD88^+/+^ mice (data not shown).

These brains were subsequently homogenized and pro- and anti-inflammatory cytokines were measured at the protein level in the supernatants. There was significantly less IFN-γ produced in MyD88^−/−^ mice compared to MyD88^+/+^ animals: 651 and 1290 pg/ml respectively (*P* <0.05) (Figure 5E). The levels of IL-12 and TNF-α were also significantly increased in the brain during *T. gondii* infection in MyD88^+/+^ compared with MyD88−/− mice (926 and 185 pg/ml respectively (*P* <0.05) for IL-12, and 57 and 37 pg/ml respectively (*P* <0.05) for TNF-α) (Figure 5E).

NO levels were not significantly different between MyD88^−/−^ mice and MyD88^+/+^ mice (5.6 and 4.1 μM respectively), and IL-13, IL-2 and IL-10 could not be detected in the supernatants (data not shown). Therefore, MyD88^−/−^ mice would have less inflammation in the brain than MyD88^+/+^ controls.

The data demonstrate highly increased susceptibility of MyD88^−/−^ mice to *Toxoplasma*-induced encephalitis, which is correlated with reduced innate and adaptive immune responses. MyD88^−/−^ mice survive the acute phase of infection, but succumb as a consequence of the presence of large numbers of parasites in the brain.

Therefore, MyD88 plays an essential role in host resistance by influencing the control of *T. gondii* replication.

## Discussion

Several *in vitro* and *in vivo* studies have shown that MyD88 is required to control the acute phase of *Toxoplasma* infection and to generate an effective T cell immune response. Currently, there is no evidence that MyD88 contributes to control the cerebral phase of infection and the MyD88-mediated mechanism controlling parasite levels in the brain remains to be defined.

We investigated the role of MyD88-dependent innate immune responses in the regulation of parasite and cell infiltration into the brain, which are a major pathogenic event of infection with *T. gondii*, by using infected BALB/c mice which develop an acute infection with little sign of diseases, either control infection and become latently infected with no clinical symptoms but form cysts in the brain as occurs in immunocompetent humans.

We report that the absence of MyD88 signaling impacts host defenses and is essential for protection during the acute stage of encephalitis of *T. gondii* infection as MyD88^−/−^ mice succumbed to *Toxoplasma* encephalitis. Conversely, no significant pathological differences were identified in other tissues distinct from the brain between MyD88^−/−^ mice and MyD88^+/+^ BALB/c mice infected with *T. gondii*, nor was there evidence of alterations in the systemic immune response as measured by levels of serum cytokines and antigen-specific IgG1 or IgG2a antibodies. This extreme susceptibility was correlated with an alteration in systemic and mucosal immune responses as measured by: 1) a low Th1 and Th2 cytokine production in the spleen and MLNs compared to the infected wild-type mice, and 2) a perturbation in chemokine transcript levels in spleens and MLN compartments. In parallel, we observed that *Toxoplasma* encephalitis, not impacted by sulfadiazine treatment, is correlated with an increased parasite burden and a lower expression of Th1-cytokine expression, especially IL-12, IFN-γ and TNF-α in the brain. Indeed a significant increase of tachyzoite and bradyzoite forms was observed in the brains of *T. gondii*-infected MyD88^−/−^ mice and it is likely that this increase was a result of a defect in an anti-toxoplasma type-1 response in the brain. It has already been observed that severe pathology in the brains of *T. gondii*-infected mice was correlated with decreased expression of type-1 mediators. It is highly probable that the decrease in proinflammatory mediators observed in the brain of *T. gondii*-infected MyD88^−/−^ mice is a direct consequence of a greater parasite burden.

IFN-γ is the major mediator of protective immunity against *T. gondii*[16] and is the absolute requirement for controlling tachyzoites proliferation during the acute stage of *T. gondii* infection, and also the central cytokine in protective immunity to the parasite and type-1 immune responses are vital for survival during infection [17-19]. The control of *T. gondii* in the CNS is dependent on local T cell production of IFN-γ, leading to the activation of anti-parasitic effector mechanisms. It has been shown that both T cells [20] and non-T cells, most likely microglia [21], need to produce IFN-γ to prevent toxoplasmosis, and that IFN-γ activates microglia and astrocytes to prevent proliferation of tachyzoites [22].

We have shown that severe toxoplasmic encephalitis in *T. gondii*-infected MyD88^−/−^ mice is associated with a marked defect in the number of CD8^+^ T cells, and a significantly greater number of CD11b^+^ and F4/80^+^ cells (but no CD11c^+^ cells) in the brain, and that resistance in infected wild-type mice is correlated with a greater number of CD8^+^ T cells and a low number of antigen-presenting cells. Therefore, it appears that recruitment of selected CD8^+^ T cells is important for the prevention of *Toxoplasma* encephalitis most likely by IFN-γ synthesis. The enhanced parasite replication and associated local decrease in the number of CD8^+^ T cells and IFN-γ production observed in the brains of MyD88^−/−^ are also noted in mice that lack inducible nitric oxide synthase (iNOS) and TNF-α signaling [23], IL-33 receptor [24] or NF-kappaB1 [25]. It also appears that trafficking of CD11b^+^ and F4/80^+^ cells to the brain is correlated to majoration of encephalitis. It was previously shown that CD11c^-^ and CD11b^+^ cells are the major cell population that disseminates the parasite to the brain, and that macrophages might be involved in carrying tachyzoites in the brain after infection [26].

We have also analyzed the production of chemokines after *T. gondii* infection at brain level. In contrast to wild-type mice we detected only low levels of chemokine mRNA in the brain of infected MyD88^−/−^ mice. Levels of CCL3, CCL5, CCL7 and CCL19, which are most abundant in the brain of infected wild-type mice, were limited in the MyD88^−/−^ mice. Regardless of a direct or indirect effect, it is clear that MyD88 is crucial for inducing expression of these four chemokines in the brain during *T. gondii* infection. However, the importance of MyD88 in chemokine expression in the brain of mice during *Toxoplasma* infection has not been reported before. It is possible that macrophages and astrocytes produce these chemokines in the brain, and that a majority of the cells producing the chemokines in the brains of infected mice would probably not be those infected by the parasite. Indeed after activation by IFN-γ, these cells are able to inhibit the intracellular proliferation of tachyzoites [27,28]. In relation to our binding, Wen *et al.*[29] have previously reported that CXCL9, CXCL10 and CCL5 are the chemokines predominantly induced in the brain of mice during *Toxoplasma* infection, and their expression is dependent on IFN-γ. Expression of CCL3, CCL5, CCL7 and CCL19 chemokines could play a crucial role in recruitment of IFN-γ-producing CD8^+^ T cells into the brain, and in facilitating migration of effector macrophages for prevention of *Toxoplasma* encephalitis during acute stage of encephalitis.

In summary, we demonstrate that during infection MyD88 signaling is essential for the development of an efficient intracerebral cellular immune response able to control parasite levels. The results presented here establish a role for MyD88 in T cell-mediated control of *T. gondii* in the CNS.

## Abbreviations

CNRS: Centre National de la Recherche Scientifique; CNS: central nervous system; DAB: 3,3’-diaminobenzidine; DC: dendritic cell; ELISA: enzyme-linked immunosorbent assay; FCS: fetal calf serum; GPI: glycosylphosphatidylinositol; HEPES: 4-(2-hydroxyethyl)-1-piperazineethanesulfonic acid; HFF: human foreskin fibroblast; i.p: intraperitoneal; IFN: interferon; IL: interleukin; iNOS: inducible nitric oxide synthase; MLN: mesenteric lymph node; NO: nitric oxide; OCT: optimal cutting temperature; PBS: phosphate-buffered saline; PCR: polymerase chain reaction; qPCR: real-time PCR; qRT-PCR: real-time reverse-transcription PCR; SEM: standard error of the mean; SPR: specific pathogen free; TLR: Toll-like receptor; TNF: tumor necrosis factor.

## Competing interest

The authors declare that they have no competing interests.

## Authors’ contribution

All authors have read and approved the final version of the manuscript.
